# Ventilation feedback device for manual ventilation in simulated respiratory arrest: a crossover manikin study

**DOI:** 10.1186/s13049-019-0674-7

**Published:** 2019-10-22

**Authors:** Abdo Khoury, Alban De Luca, Fatimata S. Sall, Lionel Pazart, Gilles Capellier

**Affiliations:** 1 Emergency Medicine Physician, Department of Emergency Medicine and Critical Care, Besançon University Hospital, Besançon, France; 2 Biomedical Research Engineer, Clinical Investigation Centre Inserm CIC-1431, Besançon University Hospital, Besançon, France; 3 Clinical Research Engineer, Clinical Investigation Centre Inserm CIC-1431, Besançon University Hospital, Besançon, France; 4 Medical Coordinator, Clinical Investigation Centre Inserm CIC-1431, Besançon University Hospital, Besançon, France

**Keywords:** Bag-valve-mask, Cardiac arrest, Manual ventilation, Ventilation feedback device

## Abstract

**Background:**

Studies have shown that providing adequate ventilation during CPR is essential. While hypoventilation is often feared by most caregivers on the scene, the most critical problem remains hyperventilation. We developed a Ventilation Feedback Device (VFD) for manual ventilation which monitors ventilatory parameters and provides direct feedback about ventilation quality to the rescuer. This study aims to compare the quality of conventional manual ventilation to ventilation with VFD on a simulated respiratory arrest patient.

**Methods:**

Forty healthcare providers were enrolled and instructed to ventilate a manikin simulating respiratory arrest. Participants were instructed to ventilate the manikin for 5 min with and without the VFD in random order. They were divided in two groups of 20 people, one group ventilating through a mask and the other through an endotracheal tube.

**Results:**

Ventilation with the VFD improved from 15 to 90% (*p* < 0.001) with the mask and from 15 to 85% (*p* < 0.001) with the endotracheal tube (ETT) by significantly reducing the proportion of hyperventilation. The mean ventilation rates and tidal volumes were in the recommended ranges in respectively 100% with the mask and 97.5% of participants with the ETT when using the VFD.

**Conclusion:**

VFD improves the performance of manual ventilation by over 70% in different simulated scenarios. By providing the rescuer direct feedback and analysis of ventilatory parameters, this device can significantly improve ventilation while performing CPR and thus save lives.

## Introduction

Sudden cardiac death is the leading cause of mortality worldwide and remains a global and serious public health problem due to its high incidence (1 per 1000 yearly) and its low survival rate (from 1 to 10% worldwide) [1–3]. Early initiation and team-focused cardiopulmonary resuscitation (CPR) increases survival, with good neurological outcomes, from 4.8 to 8.3% [3]. It focuses on optimizing chest compressions (CC) with proper compression rate and depth. It also emphasises minimal interruptions of CC and prioritizes the use of the Bag Valve Mask (BVM) with a ventilation rate of 8–10 min^− 1^. However recent studies have shown that chest compressions alone without ventilation, if prolonged, provides passive tidal volume (V_T_) lower than the estimated physiological dead space and may lead to hypoxemia [4, 5]. Providing adequate ventilation during CPR is therefore essential to maintain gas exchange for adequate carbon dioxide removal and sufficient arterial oxygen content, while minimizing the risk of impaired circulation [4].

Manual ventilation with a facemask or an endotracheal tube (ETT) is the most commonly used technique to provide ventilation during CPR. BVM ventilation is a basic airway skill mainly used by emergency medical technicians and paramedics. It has the advantage of being a quick and simple ventilation method and therefore remains the preferred method for pre-hospital care [6]. Its challenge is to maintain perfect airtightness between the mask and the patient’s face while avoiding stomach distension and pulmonary aspiration. Studies highlight failed intubation as a more common problem than failed ventilation. Epidemiologic data suggest that difficult mask ventilation occurs in 4–11% of patients in the emergency room [7]. In contrast to basic airway management, the insertion of an ETT is considered the “gold standard technique.” This, however, requires advanced skills in airway management to avoid tube misplacement or long duration of attempt, leading to excessive interruptions of chest compressions [8].

Basic and advanced airway management techniques have their own advantages and hazards. A recent study from Adnet et al. showed no difference in outcome of cardiac arrest patients ventilated with both techniques [9]. It was hypothesised that it could be due to the adverse effects associated with the inability to control ventilatory parameters with manual resuscitators [10]. While hypoventilation is feared by most of the caregivers on the scene, the most critical problem remains hyperventilation which increases intrathoracic pressures and impairs hemodynamics [11, 12]. The International Liaison Committee on Resuscitation (ILCOR) recommends ventilating cardiac arrest patients at a rate of 8 to 10 min^− 1^, and a Vt of 400–600 ml. We recently showed that caregivers, regardless of experience, tend to hyperventilate patients in 80% of cases [13]. This has also been reported in other clinical studies [11, 14].

This hyperventilation may be explained by three factors: there is no monitoring of ventilatory parameters on manual resuscitators, there is no direct evaluation of ventilation quality, and there is limited understanding of the patient’s needs from rescuers with less experience.

To address this need, we developed a Ventilation Feedback Device (VFD) for manual ventilation which monitors ventilatory parameters, estimates their target values according to patient’s need and ILCOR recommendations, and gives direct feedback on ventilation quality.

This study aims to compare conventional manual ventilation to ventilation with a VFD on the delivery of adequate ventilation. We hypothesized that the use of a VFD would help caregivers deliver adequate ventilation and thus, improve ventilation practice and reduce related risks.

## Methods

### Materials

#### Ventilation feedback device

**Fig. 1:**
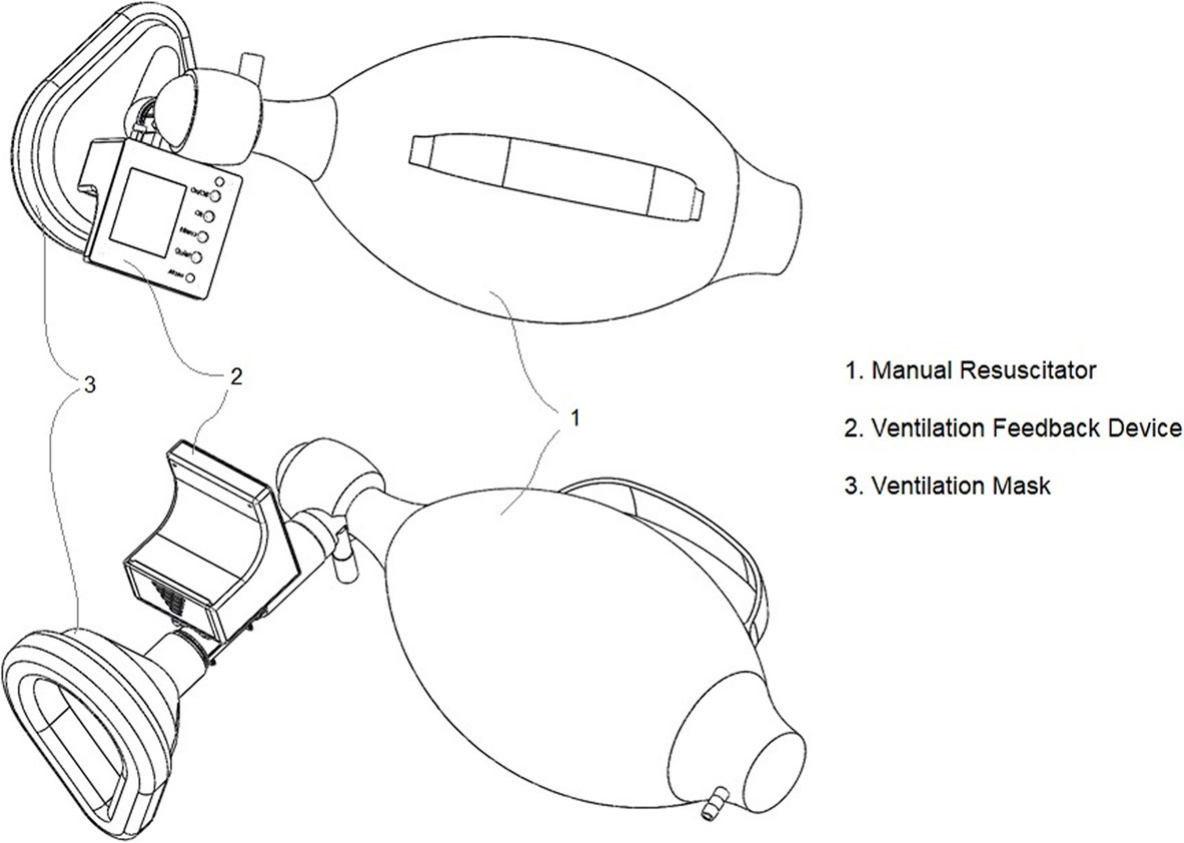
VFD plugged between a self-inflating bag (i.e. manual resuscitator) and a mask

The VFD is used to provide information on the delivery of each insufflation and to guide the ventilation through real-time feedback. It is a non-invasive monitoring device which can be plugged to any type of manual resuscitator. It is inserted between the bag and the patient interface (Fig. 1), such as a facemask, an ETT or any kind of supra-laryngeal system. The device contains a single use mass flow sensor which measures inspiratory and expiratory flows based on the principle of heat absorption. A controllable heater element is mounted in the middle of the air duct and temperature sensors are mounted symmetrically upstream and downstream from this heater element. Any air flow causes a transfer of heat which depends on the number of molecules passing through the air duct. This newly developed technology is highly sensitive and reliable, and has the advantage of avoiding water condensation and limiting dead space and airflow resistance compared to conventional pressure-gradient airflow sensors (sensor dead space < 10 mL and airway resistance = 1.8 cmH_2_O.L.s^− 1^ at 60 L.min^− 1^). It does not alter gas composition and has standard connectors and thus can be connected to any kind of capnometer to assess end tidal CO_2_.

The sensor is plugged to an electronic control unit which records and processes data to calculate the main ventilatory parameters (inspiratory/expiratory volume, tidal volume, ventilation rate, inspiratory/expiratory time, amount of leakage) at each ventilation cycle.

**Fig. 2:**
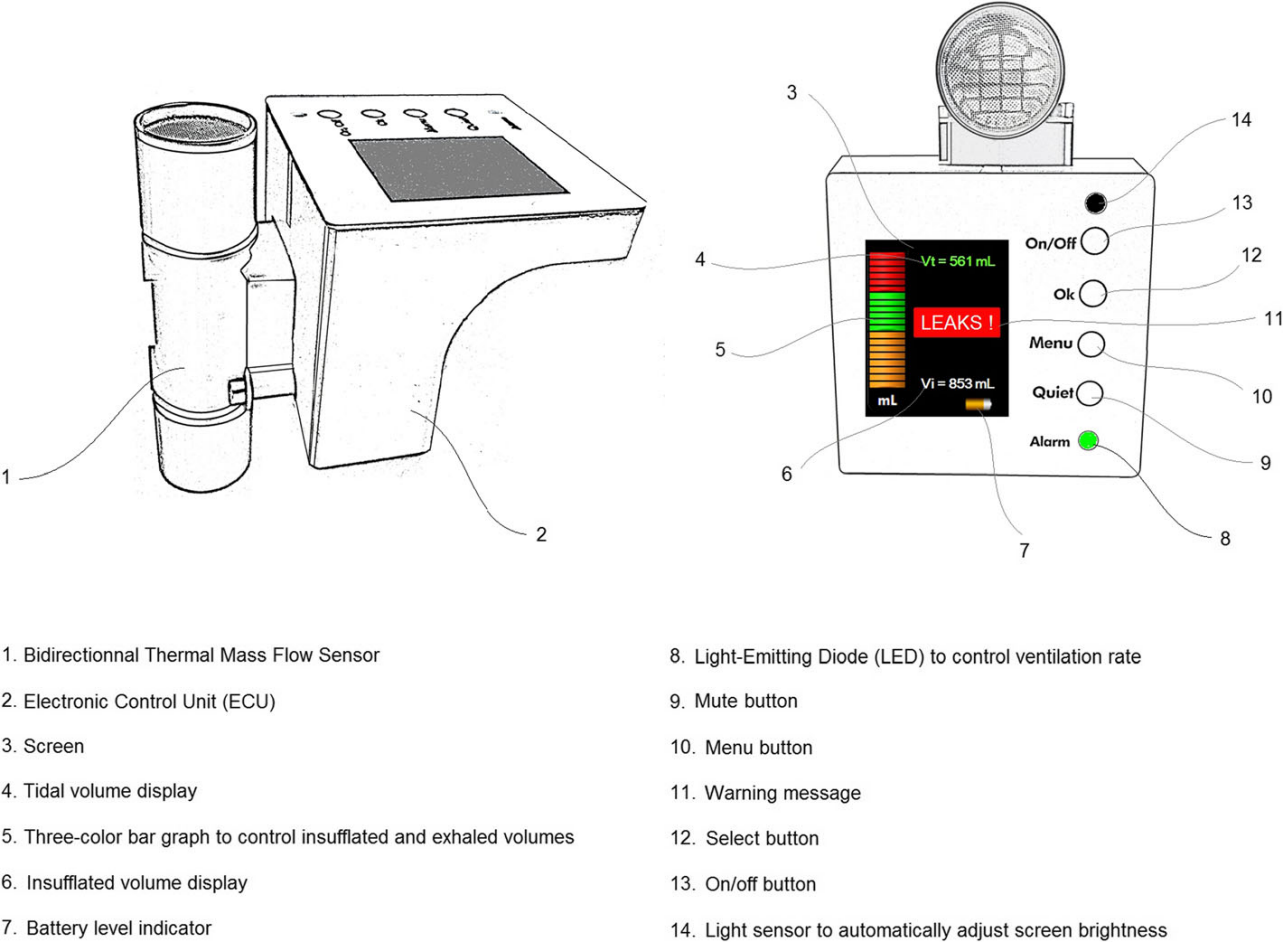
Description of the main features of the user interface of the VFD

The electronic unit has three main functions:

- *Direct feedback for ventilation rates and tidal volumes:* The FVD delivers visual and audible feedback to maintain constant and adequate ventilation. The unit displays a Bar Graph with three areas of different colours (orange for insufficient volume, green for adequate volume and red for excessive volume), which is directly correlated with the amount of air provided to the patient when the bag is squeezed (Fig. 2). When the user selects the patient’s profile, the target range of ideal V_T_ is automatically adjusted to comply with international guidelines of 6–7 mL.kg of ideal body weight (IBW).

The second visual tool is for ventilation rates. A visual signal with a green blinking light is sent to the rescuer to indicate the appropriate time to ventilate the patient (Fig. 2). This helps the user to achieve adequate ventilation rates and avoid hyperventilation.

- *Ventilation performance assessment:* The FVD makes continuous and individualized performance assessments of the ventilation being provided to the patient. It offers a comparison between target values for tidal volumes and ventilation rates and delivered values. The algorithms are also designed to assess the expiratory time constant, which depends on the airway resistance and lung compliance of the patient, in order to adjust the target ventilation rate to the unique characteristics of the patient’s lung. Finally, VFD algorithms calculate the proportion of leakage occurring during insufflation and expiration to ensure reliable estimation of V_T_.
- *Alarm system with visual and audible feedback:* The ventilation performance assessment of the VFD displays warning messages in case of inadequate tidal volumes, ventilation rates, or high level of leakage (Fig. 2).

#### Manikins

To simulate a patient in respiratory arrest, a Laerdal® Airway Management Trainer manikin (Laerdal Medical, Stavanger, Norway) was installed on a stretcher. The mannequin’s lungs were bypassed and directly connected to an ASL 5000® artificial lung (IngMar Medical, Ltd., Pittsburgh, PA, USA) to simulate an apnoeic adult patient with compliance of 70 mL.cmH_2_O^− 1^ and resistance of 3.5 cmH_2_O.L^− 1^.s. The mannequin was ventilated manually with an Ambu Spur II bag, which has a reservoir of 2600 mL (Ambu A/S Baltorpbakken 13, DK-2750 Ballerup).

### Protocol

We conducted a randomized, crossover manikin-based study with volunteers at the University Hospital of Besançon, France. They had recently received personal feedback on their practice and a brief training session on manual ventilation. Participants were divided in two groups based on their professional category and skills: fire-fighters and ambulance drivers were in the Basic Life Support (BLS) group, and emergency physicians and nurses were in the Advanced Life Support (ALS) group. Personal information and results of the participants were anonymized, and the need for ethical approval was waived by the institutional ethics committee (Comité de Protection des Personnes CPP Est II). Prior to the tests, participants signed an informed consent and fulfilled a case report form.

We asked participants to perform 5 min ventilation with and without VFD in a cross over randomization process to avoid bias. For both steps of the study (ventilation mask and tracheal tube), participants were randomly assigned to either arm (“WITH VFD”, then “WITHOUT VFD” or “WITHOUT VFD”, then “WITH VFD”) with a 1:1 ratio. Two randomization lists with blocks size of 4 were computer generated independently by the statistician team with SAS 9.4 for Windows, (SAS Institute INC., Cary, NC, USA), prior to the start of the trial and placed in numbered sealed envelopes.

There was a one-week washout period between the first test and the second test in order to limit biases related to possible learning effects. Participant from the BLS group proceeded to non-invasive ventilation with a medium adult mask, and participant from the ALS group ventilated the manikin with a tracheal tube of 8.0 mm ID.

Neither training nor written instructions about VFD was provided to the participants. However, a short oral presentation of 2 min was made to every participant before the tests.

### Ventilation performance analysis

Tidal volumes (*V*_*T*_), ventilation rates (*V*_*R*_), peak airway pressures (*P*_peak_) and inspiratory and expiratory times (*I*_time_, *E*_time_) were measured directly by the ASL 5000® lung simulator.

We aimed to evaluate the global performance of the 5-min ventilation period by considering the general tendency and time-related variability of the tidal volume and the ventilation rate. This method was tested and validated in a previous study we conducted [12]. Regarding our simulated patient model (75 kg IBW and no respiratory pathology), we considered *V*_*T*_ from 300 to 600 ml and *V*_*R*_ between 8 and 15 min^− 1^ to be acceptable for the patient. If V_*T*_ or V_*R*_ were under or over the target range, we considered the simulated patient to be hypoventilated or hyperventilated respectively.

In order to determine the accuracy of the tidal volume assessed by the VFD, we compared the V_T_ estimated by the device and those measured by the ASL 5000 for every ventilation cycle.

### Sample size estimation

In our previous work [13], we showed that manual ventilation compliant with ILCOR guidelines did not exceed 15% among the 140 healthcare professionals who participated in the study. We hypothesized that it could be improved by up to 70% with the use of VFD. A sample size of 20 healthcare professionals for each group was calculated with a power of 90% and an alpha of 5%. Subsequently, 40 participants were enrolled and divided in two groups (BLS and ALS).

### Statistical analysis

Continuous data are expressed as means ± SD. Results are presented as percentages for nominal variables. We used t-test for continuous variable including ventilation rate, tidal volume, inspiratory and expiratory time. We used paired t-test for comparing the tidal volumes measured by the VFD and by the ASL 5000®. Wilcoxon test was used to examine the difference between boxplots. Fisher exact test and McNemar test were used for comparing manual ventilation performance with and without VFD. A Bonferroni correction was applied if necessary. A *p*-value lower than 0.05 was considered to be statistically significant. Statistical analysis was performed with SAS 9.4 for Windows (SAS Institute INC., Cary, NC, USA).

## Results

Forty healthcare professionals (12 physicians, 8 nurses, 13 firefighters and 7 ambulance drivers) were enrolled into this manikin study. The mean population age was 40 ± 9 years, and 55% of them were highly experienced (professional experience ≥10 years). Nine of the volunteers were women (22.5%). The detailed characteristics of the population are shown in Table 1.

**Table 1:** Characteristics of the studied population (*n* = 40). SD = Standard Deviation

Mean age ± SD (years)	39.9 ± 8.7
Sex (*n* (%))	
Female	09 (22.5)
Male	31 (77.5)
Professional category (*n* (%))	
Physicians	12 (30.0)
Nurses	08 (20.0)
Firefighters	13 (32.5)
Ambulance drivers	07 (17.5)
Professional experience (*n* (%))	
High (≥ 10 years)	22 (55.0)
Medium (5 ≤ *n* < 10 years)	09 (22.5)
Little (< 5 years)	09 (22.5)

**Fig. 3:**
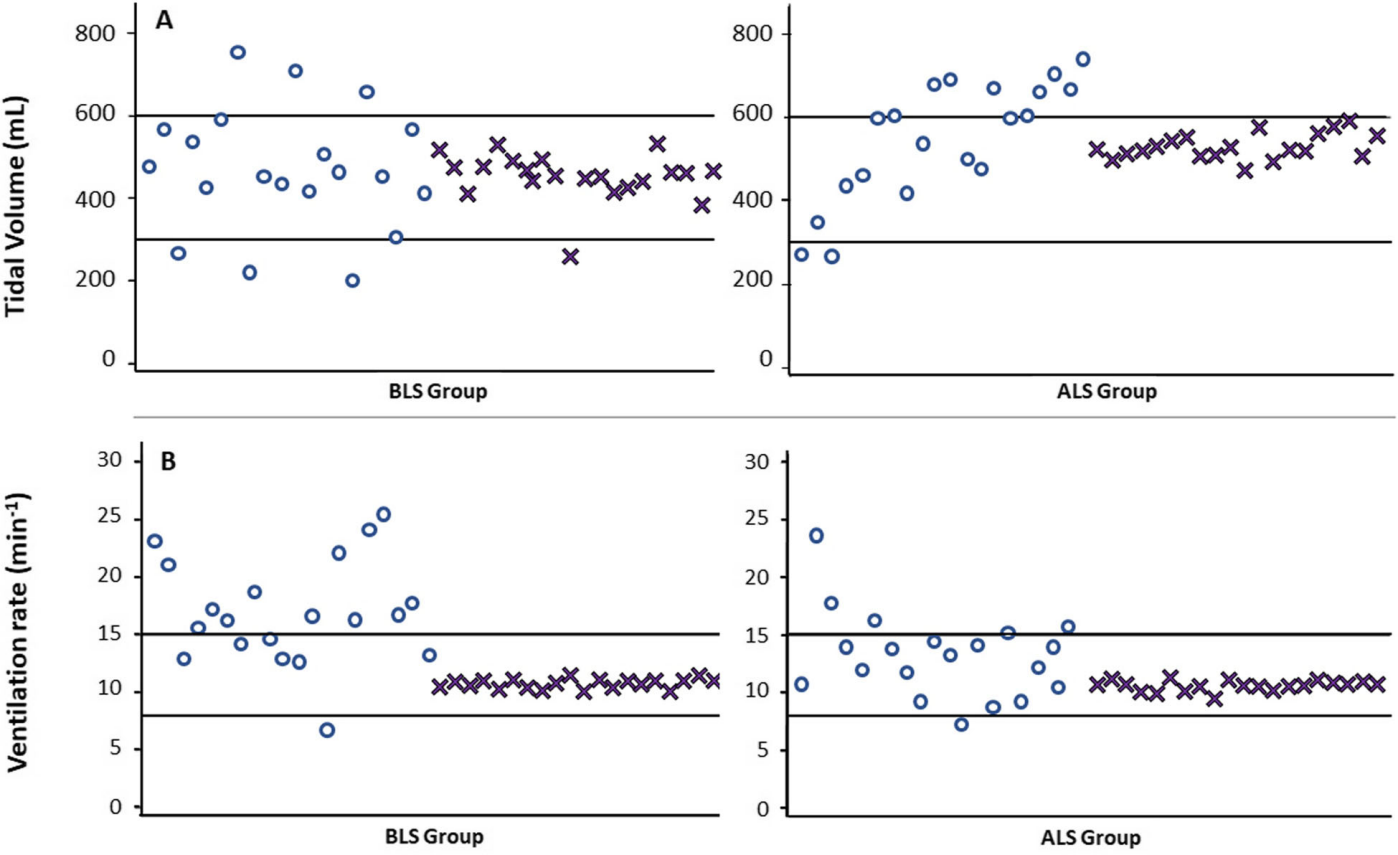
Comparison of mean tidal volume (**a**) and mean ventilation rate (**b**) for each participant between conventional ventilation () and ventilation with VFD () for BLS and ALS groups. *n* = 20 participants/group, ventilation was performed during 5 min/participant

We recorded 3029 cycles for conventional ventilation and 2083 cycles for guided ventilation with VDF. In both BLS and ALS groups, we observed a significant reduction of ventilation rates which consistently fell within the target range when ventilating with VFD, and an important decrease of tidal volume dispersion (Table 2 and Fig. 3).

**Table 2:** Comparison of ventilatory parameters between conventional manual ventilation and guided ventilation with VFD in the ALS and BLS groups (mean ± SD)

Variable	Conventional ALS group (*n* = 1382 cycles)	VFD ALS group (*n* = 1030 cycles)	*p*-value	Conventional BLS group (*n* = 1647 cycles)	VFD BLS group (*n* = 1053 cycles)	*p*-value
Ventilation rate (min^− 1^)	16.2 ± 6.9	10.7 ± 1.1	< 0.001	18.2 ± 5.0	10.8 ± 1.1	< 0.001
Tidal volume (ml)	549 ± 153	529 ± 43	< 0.001	471 ± 155	451 ± 86	< 0.001
Inspiratory time (s)	1.2 ± 0.4	1.3 ± 0.5	< 0.001	1.2 ± 0.3	1.3 ± 0.5	< 0.001
Expiratory time (s)	3.1 ± 1.5	4.3 ± 0.5	< 0.001	2.4 ± 1.0	4.2 ± 0.6	< 0.001
I/E ratio	0.5 ± 0.2	0.3 ± 0.1	< 0.001	0.6 ± 0.2	0.3 ± 0.3	< 0.001
Peak airway pressure (cmH_2_O)	10.7 ± 4.0	8.9 ± 1.1	< 0.001	8.8 ± 2.9	7.6 ± 1.6	< 0.001

**Fig. 4:**
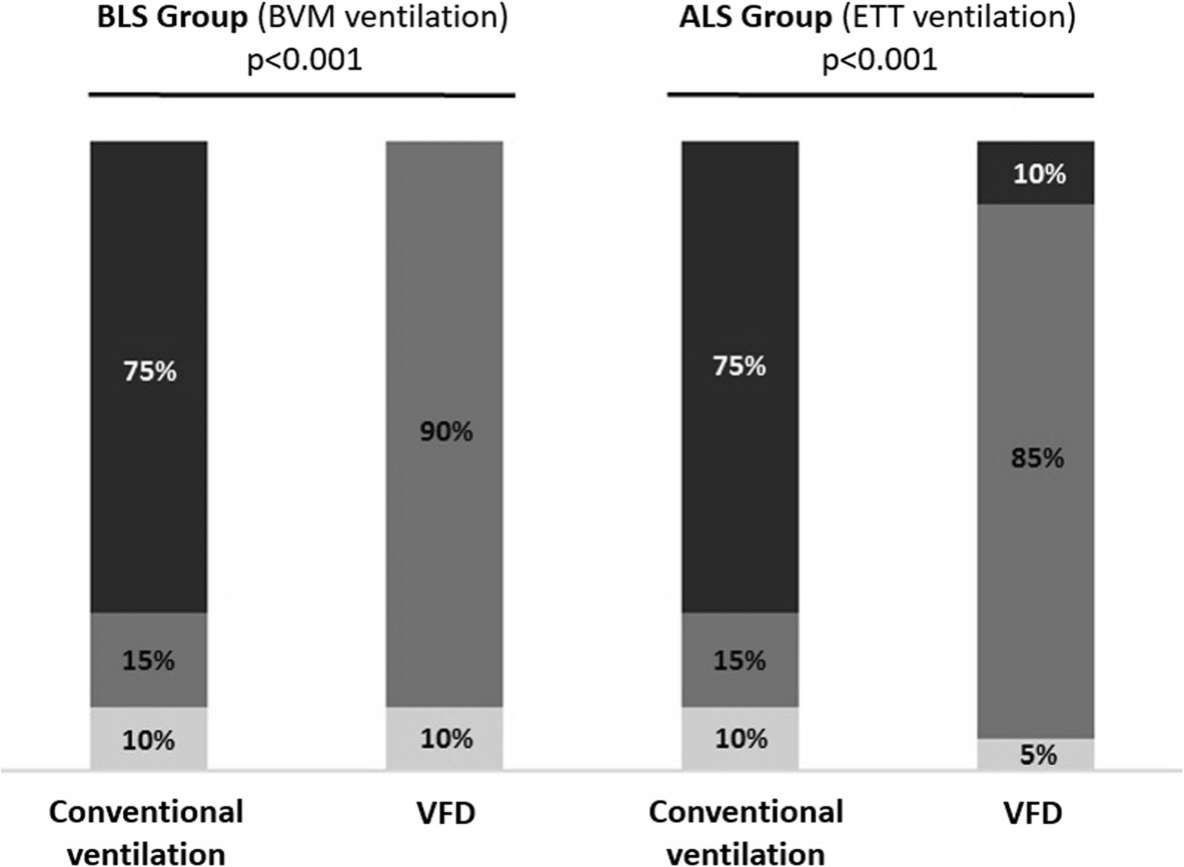
Comparison of the proportion of hypoventilation (), adequate ventilation () and hyperventilation ()between conventional manual ventilation and guided ventilation with VFD in the BLS and ALS groups (*n* = 40 participant

By analysing the performance of the 5-min ventilation sequences, we found an improvement of ventilation performance with the use of VFD compared to conventional ventilation (Fig. 4). Ventilation improved from 15 to 90% (*p* < 0.001) in the BLS group, and from 15 to 85% (*p* < 0.001) in the ALS group by significantly reducing the proportion of hyperventilation.

Regarding the accuracy of the tidal volume assessed by the VFD when compared with those measured by the ASL 5000, we found a mean deviation of only − 2.62 ± 8.80 ml when ventilation was provided with a mask in the BLS group (*p* < 0.001), and − 0.12 ± 4.48 ml in the ALS group (*p* = 0.40).

## Discussion

When using the newly developed Ventilation Feedback Device, we were able to show a significant improvement in manual ventilation quality. Both experienced and less experienced basic and advanced caregivers improved their ventilation. The mean ventilation rates and tidal volumes were in the recommended range in respectively 100 and 97.5% of the simulated ventilation sequences.

The use of VFD also reduced the mean ventilation frequency from 18.2 ± 5.0 to 10.8 ± 1.1 min^− 1^ in the BLS group (*p* < 0.001), and from 16.2 ± 6.9 to 10.7 ± 1.1 min^− 1^ in the ALS group (p < 0.001). Even more important, this new device eliminates the inter-individual variations in performance with very low dispersion of ventilation frequencies and tidal volumes as compared to conventional manual ventilation (Fig. 3). The new algorithms developed in VFD also proved to be highly reliable in the assessment of the tidal volume even in the presence of significant leakage, with a mean deviation of only − 2.62 ± 8.80 ml in the BLS group compared to the values measured by the ASL5000.

The analysis of the 5-min ventilation sequences reveals that only 15% of participants provided adequate ventilation and 75% hyperventilated the simulated patient in both BLS and ALS groups (Fig. 4). The use of VFD improved ventilation from 15 to 90% in the BLS group, and from 15 to 85% in the ALS group. It is noteworthy that none of the participants received written instructions or training with the device before performing ventilation: 97.5% of them found the device intuitive and useful in their future practice.

Our results confirm the need for VFD. Indeed, the literature shows that despite adequate training and in-depth experience, professional rescuers consistently hyperventilate patients during CPR [11, 14, 15]. The absence of feedback on ventilatory parameters could be the main explanation, and Bowman et al. recently showed that visualizing the insufflated volumes resulted in an improvement of 47% in ventilation performance [16].

Recently, many attempts have been made to improve manual ventilation quality. Nehme et al. tried to optimize the mechanical size and shape of manual resuscitators and showed that inadequate tidal volumes and rates fell by 27 and 23% respectively; however, it still resulted in 70% inadequate ventilation [17]. Lim et al. showed that using a modified BVM with audible metronome function allowed emergency care personnel to deliver more constant ventilation rate, but it did not help in identifying leakage, hypoventilation or inadequate tidal volumes [18].

Also recently, the German company Weinmann Emergency™ advanced a manually triggered ventilation device, the Easy CPR® device, to replace manual resuscitators. This device, a mix between manual and mechanical ventilation, was heavy weighted, complex to use and not very ergonomic. It brought many expectations but failed to convince. Bergrath et al. found that the Easy CPR® was not advantageous in the setting of CPR and carried a risk of prolonged no-flow time [19]. BVM was also rated as easier to use by rescuers.

Marjanovic et al. however suggested that the Easy CPR® may improve ventilation and decrease the risk of pulmonary overdistention but failed to show significant improvement in tidal volume delivery: 25.6% of ventilations fell in the required 400–600 mL range using manual BVM ventilation, and only 3% more (28.6%) using the Easy CPR® [ 20].

In view of the recently developed devices and techniques that have not significantly improved performance, the VFD offers an important solution to avoid hyperventilation and improve manual ventilation quality in pre-hospital care.

Recently, important advances have been made in CPR feedback devices and have demonstrated a real capacity to optimize chest compressions [3, 21–24]. Unfortunately, improvements in manual ventilation quality have been overlooked. There is now substantial clinical evidence that hyperventilation reduces coronary perfusion pressure [11, 25, 26] and induces cerebral vasoconstriction due to a drop of carbon-dioxide partial pressure [27, 28]. Aufderheide et al. have shown in a porcine cardiac arrest model that a reduction of ventilation rates from 30 to 12 min^− 1^ increased the survival rate from 14 to 68% [26]. Our findings therefor have important implications for the successful performance of CPR. Our device shows promise for improving cardiac-arrest survival done by first responders and caregivers.

This study supports the superior technical performance of VFD and its ability to guide healthcare professionals in delivering adequate ventilation, but it has some limitations. Ventilations were performed on a simulated “easy to ventilate” patient which cannot reproduce heart-lung interactions during CPR. In real-life practice passive ventilation generated by the chest compressions may affect the measurement and interpretation of the ventilatory parameters. The VFD algorithms have been developed with an adjusted trigger to filter the artefacts caused by passive ventilation and chest movements. Nonetheless, it needs to be tested on humans to ensure it performs as well. Further clinical investigations are needed to determine the extent of the clinical benefits which might be provided by such a device.

## Conclusion

VFD has proven its ability to improve the performance of manual ventilation by more than 70% while avoiding the risks of hyperventilation in different simulated scenarios. With direct feedback and analysis of ventilatory parameters, this device allows the healthcare professional to adhere to the ILCOR guidelines while providing manual ventilation. The new sensing technology was reliable and intuitive and might have important implications for the management of cardiac arrest patients in the near-future.

## Data Availability

Restrictions apply to the availability of these data, which were used to develop the VFD for the current study, and so are not publicly available.
